# Efficacy analysis of the Tendvia™ pulmonary artery stent thrombectomy system in the treatment of intermediate- to high-risk pulmonary embolism

**DOI:** 10.3389/fcvm.2025.1685294

**Published:** 2025-10-14

**Authors:** Xunlai Lu, Shengfu Liu, Changmei Chen, Junwen Chen, Shenrui Xu, Yuanjia Luo, Jiachao Luo

**Affiliations:** ^1^ Department of Cardiovascular Surgery, Northeast Yunnan Central Hospital, Zhaotong, China; ^2^ Department of Cardiovascular Medicine Ward 3, Northeast Yunnan Central Hospital, Zhaotong, China

**Keywords:** pulmonary embolism, troponin, high-risk group, percutaneous mechanical thrombectomy, pulmonary artery pressure

## Abstract

**Objective:**

To investigate the clinical efficacy of the Tendvia™ pulmonary artery stent thrombectomy system in treating patients with intermediate-high risk and high-risk acute pulmonary embolism.

**Methods:**

A total of 15 consecutive patients with acute pulmonary embolism (classified as intermediate-high risk and high risk) admitted to Northeast Yunnan Central Hospital from January 2025 to March 2025 were enrolled. Among them, there were 7 male and 8 female patients, with a mean age of (65.2 ± 11.38) years. All patients underwent percutaneous mechanical thrombectomy (PMT) using the Tendvia™ pulmonary artery stent thrombectomy system. General patient information, including history of venous thromboembolism (VTE), surgical history within four weeks, and embolism locations, was recorded. Changes in pulmonary artery pressure before and after thrombectomy, as well as preoperative and postoperative ratios of right ventricular diameter to left ventricular diameter (RV/LV) on echocardiography, and relevant results from biochemical tests such as troponin, D-dimer, and N-terminal pro-B-type Natriuretic Peptide (NT-BNP), were compared. The improvement in right heart function, hemodynamic changes, and alterations in myocardial enzymes were evaluated to assess the safety and efficacy of the Tendvia™ pulmonary artery stent thrombectomy system**.**

**Results:**

The surgical procedures were effective in all 15 patients, with no mortality reported. Pulmonary artery pressure significantly decreased after thrombectomy compared to preoperative levels, demonstrating statistical significance. Symptoms such as chest tightness, chest pain, and dyspnea were markedly alleviated in all patients.

**Conclusion:**

The Tendvia™ pulmonary artery stent thrombectomy system demonstrates definitive efficacy in treating intermediate-high risk and high-risk pulmonary embolism, with a low incidence of surgical complications.

## 1 Introduction

The incidence of pulmonary embolism (PE) has been on the rise in recent years, with pulmonary thromboembolism (PTE) being the most prevalent type. Its primary pathological alterations include hemodynamic changes, right ventricular dysfunction, and respiratory function impairment, making it a common life-threatening cardiovascular disease. Early diagnosis and treatment can significantly improve patient prognosis. The Tendvia™ pulmonary artery stent thrombectomy system, independently developed in China, employs a design principle similar to that of the American FlowTriever thrombectomy system. It utilizes a large-bore aspiration catheter combined with a unique disc-shaped basket-type thrombectomy device to remove thrombi. This study retrospectively analyzed the efficacy and safety of the Tendvia™ pulmonary artery stent thrombectomy system in surgical treatment of 15 patients with intermediate-high risk and high-risk acute pulmonary embolism (APE), providing insights into percutaneous mechanical thrombectomy (PMT) for the management of APE.

## 2 Subjects and methods

### 2.1 Study subjects

This retrospective, single-center study consecutively enrolled 15 patients who were admitted to Northeast Yunnan Central Hospital from January 2025 to March 2025, diagnosed with acute pulmonary embolism (APE), and underwent percutaneous mechanical thrombectomy (PMT) using the Tendvia™ pulmonary artery stent thrombectomy system. All patients signed informed consent for the surgical procedure, including 7 males and 8 females.

Inclusion criteria:

Classified as intermediate-high risk APE according to the 2019 European Society of Cardiology guidelines for risk stratification of pulmonary embolism;
Onset time of PE-related symptoms ≤14 days;
Simplified Pulmonary Embolism Severity Index (sPESI) score ≥1;
Right ventricular/left ventricular maximum diameter ratio ≥0.9 as measured by CT pulmonary angiography (CTPA).

Exclusion criteria:

Onset time of pulmonary embolism symptoms >2 weeks;
Intolerance to interventional treatment;
Patients with coagulopathy and clinically significant bleeding risk;
Severe liver or renal dysfunction;
Patients with active malignancy;
Expected survival <6 months.

### 2.2 Selection of surgical consumables

The surgical consumables selected for the procedure included:One 5F vascular sheath set (Terumo) One 11F vascular sheath set (Terumo) One Proglide vascular closure device (Abbott) One 0.035*260 cm angled guidewire (Terumo) One 5F*125 cm MPA catheter (Codis) One 5F*110 pigtail catheter (Eptech) One 0.035*260 cm Amplatz guidewire (Boston Scientific) One 22F (or 18F) vascular sheath (Tendvia, China) One set of Tendvia™ pulmonary artery stent thrombectomy system.

### 2.3 Treatment process

All patients underwent comprehensive preoperative evaluations, including echocardiography, CTPA, lower extremity vascular ultrasound, blood routine tests, NT-proBNP, D-dimer, and myocardial enzyme profile. CTPA confirmed the presence of filling defects in at least one main pulmonary artery or proximal lobar artery, while echocardiography indicated signs of right ventricular overload or myocardial enzyme alterations. All patients underwent PMT using the Tendvia™ stent thrombectomy system via a right femoral vein approach. Four patients underwent the procedure under general anesthesia with tracheal intubation, while the remaining patients received local anesthesia in the right inguinal region. Twelve patients had concurrent peripheral deep vein thrombosis (DVT), and two patients had a surgical history within the past four weeks. Intraoperative vital signs were continuously monitored.

The surgical procedure was as follows: Patients were placed in a supine position. After satisfactory anesthesia, the right common femoral vein was punctured using the Seldinger technique, and a 5F vascular sheath was inserted. A pre-placed suture device (Abbott) was prepared, followed by replacement with an 11F vascular sheath and systemic half-dose heparinization. Under C-arm fluoroscopy, a 260 cm hydrophilic guidewire was advanced in conjunction with a 5F pigtail catheter through the inferior vena cava-right atrium-right ventricle into the main pulmonary artery. The guidewire was withdrawn, and high-pressure angiography was performed to assess the extent of pulmonary embolism and localize the thrombus. The guidewire was reintroduced, and a 5F MPA catheter was advanced into the main pulmonary artery. A pressure-measuring extension tube was connected to measure and record pulmonary artery pressure.A 260 cm hydrophilic guidewire was then inserted, and a 5F MPA1 catheter was used to superselect a thrombosed pulmonary artery segment on one side. The guidewire was exchanged for a Boston Scientific 260 cm Amplatz guidewire, the 11F short sheath was withdrawn, and a Tendvia™ 22F (or 18F) long sheath was inserted. A 20F (or 16F) Tendvia™ thrombectomy sheath was advanced through the long sheath, and negative pressure aspiration was performed twice using a 50 ml syringe. After aspiration, a 15–18 mm (or 11–14 mm) thrombectomy stent was introduced to retrieve the thrombus once, followed by another two rounds of negative pressure aspiration using a 50 ml syringe. The same procedure was repeated for the thrombosed segment on the contralateral side.

**Figure 1:**
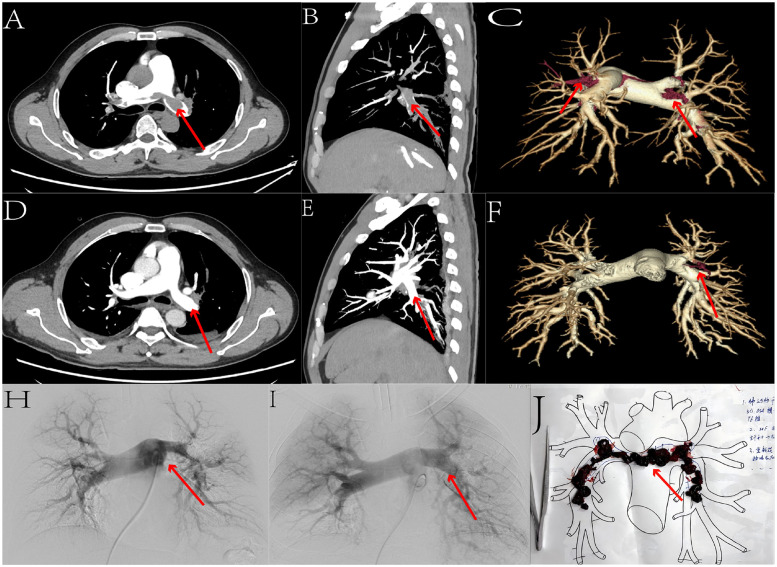
We selected the data from one patient. Figures **(A,D)** present axial comparisons of the percutaneous pulmonary artery thrombectomy before and after the procedure. Figures **(B,E)** show sagittal comparisons, while Figures **(C,F)** display three-dimensional reconstruction comparisons. Figures **(H,I)** illustrate angiographic comparisons before and after the surgery. Through these sets of comparative images, we can clearly observe a significant reduction in pulmonary artery thrombi, with marked recanalization of the occluded pulmonary arteries. Figure **(J)** depicts the freshly extracted thrombus.

After aspiration, a 5F pigtail catheter was exchanged to assess the thrombectomy effect via angiography. Subsequently, 200,000 units of urokinase dissolved in 20 ml of normal saline were pulsatile-injected through the pigtail catheter. A 260 cm angled guidewire was introduced, the thrombectomy sheath was withdrawn, and a 5F MPA catheter was advanced into the main pulmonary artery. The pressure-measuring extension tube was reconnected to measure pulmonary artery pressure again (Figure 1). Postoperatively, all patients experienced relief of symptoms such as chest pain and dyspnea.

### 2.4 Follow-up methods

All patients were followed up for at least three month via outpatient visits or telephone calls, during which changes in symptoms, physical signs, and special examination results were recorded, and medication adjustments were made accordingly.

### 2.5 Statistical analysis

Data were analyzed using SPSS 29.0 statistical software package. Measurement data conforming to a normal distribution were expressed as mean ± standard deviation (x ± s), while categorical data were presented as frequencies (percentages). Measurement data that did not follow a normal distribution were described using the median (upper and lower quartiles). Comparisons of preoperative and postoperative data were conducted using paired-sample *t*-tests or paired Wilcoxon signed-rank tests, as appropriate. A *P*-value < 0.05 was considered statistically significant.

## 3 Results

The general conditions of the patients are shown in Table 1.

**Table 1:** General information of 15 patients.

Items	Numerical values (*N* = 15)
Male	7 (46.7%)
Female	8 (53.3%)
Age	65.2 ± 11.38
Age (>65 years old)	10 (67.78%)
History of VTE (Venous Thromboembolism)	12 (80%)
History of malignant tumor	1 (0.06%)
History of surgery within the past four weeks	2 (13.33%)
Hypertension	4 (26.66%)
Diabetes	2 (13.33%)
Coronary heart disease	0
High-risk group	4 (26.66%)
Moderate-to-high-risk group	11 (73.33%)
Embolism site	
Bilateral pulmonary arteries	10 (67.78%)
Unilateral pulmonary artery	5 (33.33%)

Surgical results are shown in Table 2.

**Table 2:** Surgical conditions (*n* = 15).

Items	Preoperative	Postoperative	T/Z-value	*P*-value
Heart Rate (beats/min)	81 (73,100)	77 ± 12	−2.27	0.023
Lactic Acid (mmol/L)	1.21 (1,1.6)	1.18 (1,3）	−0.15	0.875
D-dimer (mg/L)	4.37 (3.23,7.29)	5.74 (2.17,7.45)	−0.34	0.733
NT-proBNP (ng/L)	14,590.33 ± 9,351.06	5,385.00 ± 3,547.78	5.37	<0.001
Troponin (ng/ml)	296.36 (210.36,664.72)	171.16 (91.5,456.98)	−0.682	0.496
Blood Gas PO2 (mmHg)	86.94 ± 23.12	116.02 ± 27.40	−4.37	<0.001
Intraoperative Pulmonary Artery Systolic Pressure (mmHg)	50.07 ± 11.96	28.93 ± 7.84	9.425	<0.001
Intraoperative Pulmonary Artery Diastolic Pressure (mmHg)	34.60 ± 7.86	19.80 ± 7.72	9.012	<0.001
Miller index	0.45 ± 0.08	0.12 ± 0.06	18.94	<0.001
Blood Loss (ml)	193.33 ± 65.10			
Duration of Surgery (min)	141.47 ± 25.30			

All patients returned to the hospital for follow-up examinations, including echocardiography and CTPA, three months after discharge. The assessments focused on evaluating the mean pulmonary artery pressure and Qanadli index (1). The results showed that patients' symptoms were significantly alleviated compared to those before surgery, and right ventricular function had markedly improved.Cardiac ultrasound follow-up data for 3 months are shown in Table 3.

**Table 3:** Three-month follow-up data from echocardiography and CTPA (*n* = 15).

Items	Preoperative	3 month postoperative	*t*-value	*P*-value
Mean pulmonary artery pressure (mmHg);	59.27 ± 10.47	34.43 ± 8.09	12.504	<0.001
RV/LV	1.02 ± 0.11	0.56 ± 0.10	12.957	<0.001
Qanadli index	15.13 ± 3.35	2.27 ± 1.83	13.26	<0.001

## 4 Discussion

Pulmonary thromboembolism (PTE) represents the most severe manifestation of venous thromboembolism (VTE), characterized by high morbidity and mortality rates. It can present with symptoms such as dyspnea, pleuritic chest pain, hemoptysis, tachycardia, and hypoxemia, or manifest as severe hemodynamic compromise, including sudden death, shock, syncope, and other critical conditions (2). It is one of the three leading fatal cardiovascular diseases in clinical practice. The most crucial factor determining the prognosis of pulmonary embolism (PE) is the impact of PE on the right ventricle (RV) and the RV's response to it (3). Based on indicators such as hemodynamics, myocardial enzyme levels, and right ventricular function, patients are classified into low-risk, intermediate-risk, and high-risk groups (4). The 2019 European Society of Cardiology (ESC) Guidelines for the Diagnosis and Management of Acute Pulmonary Embolism recommend immediate systemic thrombolytic therapy for most high-risk patients (5).Due to risks such as hemodynamic instability or right ventricular dysfunction, active treatment is required to improve prognosis. In recent years, the incidence of pulmonary embolism (PE) has gradually increased, and endovascular therapies have advanced rapidly. Endovascular interventional techniques, including catheter-directed thrombolysis (CDT), percutaneous mechanical thrombectomy (PMT), inferior vena cava filter placement, and percutaneous mechanical fragmentation of thrombi, are increasingly being applied in clinical practice (6). Satisfactory therapeutic outcomes have been achieved. In this cohort of 15 intermediate-to-high-risk patients, the Tendvia± pulmonary artery thrombectomy stent system was selected for percutaneous mechanical thrombectomy (PMT) in all cases. The procedures were performed successfully, with patients demonstrating improved hemodynamic stability compared to preoperative levels and significant enhancement of right ventricular function postoperatively.

Endovascular therapy has gradually become an important treatment option for intermediate-to-high-risk patients with pulmonary embolism in recent years (7). Among these techniques, catheter-directed thrombolysis (CDT) involves using a super-smooth loach guidewire or a stiff guidewire in combination with a pigtail angiographic catheter to achieve pulmonary artery trunk angiography, thereby precisely identifying the site of obstruction in the pulmonary artery trunk or its branches. The catheter tip is then advanced into the thrombus segment, enabling direct infusion of thrombolytic agents—typically urokinase—through the catheter, which allows for achieving a high local drug concentration within the targeted area (8). However, this regional drug delivery strategy cannot completely eliminate the risk of bleeding complications associated with thrombolytic agents, and some patients may be ineligible for catheter-directed thrombolysis due to contraindications. Percutaneous mechanical fragmentation employs specialized catheters with uniquely shaped and rigid tips to break up emboli in the pulmonary artery trunk and its larger branches, allowing the fragmented microthrombi to be carried by blood flow to the distal branches of the pulmonary arteries (9). It can rapidly restore pulmonary artery blood flow, reduce pulmonary artery pressure and right ventricular load, increase cardiac output, and stabilize cardiopulmonary hemodynamics. However, it does not completely clear the thrombus. The drawbacks of percutaneous mechanical fragmentation are evident: it can only fragment thrombi in large pulmonary vessels and cannot remove them. The fragmented thrombi reaching the pulmonary capillary beds may induce reactive spasm of the peripheral pulmonary arteries, resulting in generally modest effects in unblocking the pulmonary arteries and reducing pulmonary artery pressure. Percutaneous mechanical thrombectomy (PMT) represents a novel thrombus treatment technique applied under such circumstances. It has been proven to reduce thrombus burden and pulmonary artery pressure while improving right ventricular function in patients with high-risk or intermediate-to-high-risk pulmonary embolism. PMT offers advantages such as a short learning curve, minimal surgical trauma, rapid recovery, and fewer postoperative complications.

The FlowTriever system from the United States has been demonstrated to be safe and effective when used in intermediate- and high-risk patient groups (10). However, during the treatment of patients in this cohort, the FlowTriever system was not yet available in the Chinese market. Currently, mechanical thrombectomy devices such as the Indigo Thrombectomy System, AngioJet Rheolytic Thrombectomy Catheter, and AcoStream Thrombus Aspiration Catheter are more widely used in China and have been clinically applied in some single-center studies. Nevertheless, large-scale, multicenter clinical trials are still lacking. The AngioJet system is capable of removing large thrombi and rapidly restoring blood flow, but its mechanical disruption may cause hemolysis, leading to hemoglobinuria and, in severe cases, acute kidney injury (11, 12). The Indigo Thrombectomy System is a relatively small aspiration catheter connected to a continuous vacuum pump, which utilizes vacuum suction to remove thrombi, demonstrating favorable clinical outcomes. Studies by Sista et al. have shown that when used for pulmonary embolism, the Indigo system resulted in a 27.3% reduction in the right ventricular/left ventricular (RV/LV) ratio between preoperative and 48-hour postoperative assessments, with 98.3% of patients avoiding intraoperative thrombolysis and an adverse event rate of only 1.7%. Due to its smaller catheter diameter, the system offers relatively easy manipulation and distal control. However, it cannot clear intact thrombi through sudden convective forces, and the smaller catheter may create channels within the thrombus rather than fully removing it (13, 14). The AcoStream thrombus aspiration device comprises a negative-pressure suction pump and a thrombus aspiration catheter, primarily used for the clinical treatment of pulmonary thromboembolism and deep vein thrombosis of the lower extremities, with applications also reported in acute superior mesenteric artery embolism (15). The AcoStream thrombus aspiration device does not rely on thrombolysis as its foundation. Clinical studies have demonstrated significant postoperative symptom improvement in patients. However, hemoglobin levels at 72 h post-procedure were notably lower compared to preoperative levels. Therefore, when using the AcoStream device for pulmonary artery aspiration in patients with severe preoperative anemia, careful attention should be paid to the volume of bleeding. Additionally, there may be instances where large thrombi obstruct the catheter, making aspiration difficult.

Systemic thrombolysis in patients with high-risk pulmonary thromboembolism (PTE) can reduce the risk of acute right heart failure, all-cause mortality, and recurrence rates (16). However, considering the higher bleeding-related risks in the Asian population, the application of thrombolytic therapy is restricted to specific patients. With the continuous advancement of interventional techniques and devices, the scope of endovascular treatment has become increasingly broad. In this patient cohort, all individuals were treated using the domestically developed Tendvia™ pulmonary artery thrombectomy system (Shanghai Tenvia Medical Technology Co., Ltd., China). Through the femoral vein approach, a large-bore aspiration catheter combined with a disc-shaped mesh basket was employed for synchronous negative-pressure aspiration and thrombectomy, ensuring efficient thrombus removal. This method simultaneously avoided the formation of small thrombi due to catheter-directed thrombolysis or fragmentation therapy. Additionally, it minimized the risk of major bleeding associated with thrombolytic agents.

This study utilized the Tendvia™ thrombus aspiration system to treat 15 patients with intermediate-to-high-risk pulmonary embolism. A retrospective analysis was conducted to compare preoperative and postoperative parameters, including mean pulmonary artery pressure (mPAP), right ventricular/left ventricular (RV/LV) ratio, and arterial oxygen partial pressure (PaO_2_). Relevant intraoperative indicators, such as procedure duration and blood loss volume, were also evaluated. All patients demonstrated favorable clinical outcomes, with immediate thrombus removal graded as Class III in 8 cases and Class II in 7 cases, indicating satisfactory thrombus clearance. Postoperatively, symptoms such as chest pain and dyspnea were alleviated. One patient developed a puncture site hematoma, which resolved after local compression. Two patients experienced significant intraoperative blood loss but recovered following postoperative blood component transfusion. No catheter-related vascular or cardiac injuries were observed in any patient. Intraoperative real-time pulmonary artery pressure monitoring indicated effective thrombectomy, with a significant reduction in the Miller index compared to preoperative levels (*p* < 0.001) (17). Postoperatively, patients' oxygen requirements decreased markedly. Three months later, follow-up echocardiography revealed significant improvement in right ventricular function, and CTPA demonstrated a notable decline in the Qanadli index (*p* < 0.001).

In the initial phase of application, we opted for compression hemostasis at the puncture site, but the hemostatic effect was uncertain. Through accumulated experience, we switched to using the Abbott vascular closure device to close the puncture site in a pre-positioned manner. After withdrawing the sheath, the suture knot was tightened, and usually, one closure device was sufficient to achieve satisfactory hemostasis. For pulmonary artery trunk angiography, we selected the DSA mode with the following parameter settings: flow rate of 18 ml/s, injection volume of 20 ml, and pressure limit of 600 psi. However, satisfactory angiographic results required patient breath-holding, which was challenging for some patients. For patients under general anesthesia with tracheal intubation, clear and stable images could be obtained by coordinating with apnea. During the process of advancing a 260 cm loach guidewire along with an MPA catheter or pigtail catheter to the pulmonary artery trunk, there was a possibility that the guidewire might pass through the tricuspid valve and simultaneously penetrate the chordae tendineae without being detected. Given the thin diameter of the guidewire and catheter, the resistance during advancement was minimal. However, when switching to a stiff guidewire (Amplatz) for advancing the thrombectomy sheath, significant resistance might be encountered at the tricuspid valve. In such cases, it was advisable not to forcefully advance the guidewire and catheter but to completely withdraw them and re-establish access. Ensuring a smooth and unobstructed advancement process was crucial for surgical safety. If advancing the guidewire and catheter was difficult despite establishing access, and advancing the delivery sheath was also challenging, but abandoning the established access was undesirable, an 8 mm fully inflated balloon could be advanced over the guidewire. If the balloon advanced smoothly, it indicated that the chordae tendineae had not been traversed, ensuring safe advancement. During thrombus aspiration, we advanced the thrombectomy sheath so that the sheath core reached the starting point of the thrombus. Then, we loosened the sheath core thread, kept the sheath core fixed, and continued advancing the thrombectomy sheath to the tip of the sheath core. Subsequently, we withdrew the sheath core. This method allowed the thrombectomy sheath to advance an additional 1 cm forward while avoiding direct insertion of the sheath into the thrombus, which could cause distal thrombus displacement. For thrombi in the left and right main pulmonary arteries, we typically performed two aspirations followed by one thrombus retrieval and then two more aspirations. For thrombi in the first-order branches, we usually followed a sequence of two aspirations, one thrombus retrieval, and one more aspiration. For patients with unsatisfactory results after the first two thrombectomy attempts, injecting 200,000 units of urokinase directly onto the thrombus before thrombus retrieval and allowing it to sit for 10 min before proceeding with retrieval was a viable option.

## 5 Conclusion

This retrospective study demonstrated that percutaneous pulmonary artery thrombectomy using the Tendvia™ stent-retriever thrombectomy system is safe, enabling immediate reduction in thrombus burden, lowering pulmonary artery pressure, alleviating symptoms, and significantly improving right ventricular function. However, this study has certain limitations, including its single-center design, small sample size, short follow-up duration, and inherent biases associated with retrospective studies, which may introduce potential confounding factors and errors. Subsequent multicenter prospective cohort studies with larger sample sizes and extended follow-up periods are warranted to comprehensively evaluate the efficacy and safety of the Tendvia™ stent-retriever thrombectomy system.

## Data Availability

The original contributions presented in the study are included in the article/Supplementary Material, further inquiries can be directed to the corresponding author.
